# Diaqua­bis­(2-hy­droxy-5-meth­oxy­benzoato-κ*O* ^1^)zinc

**DOI:** 10.1107/S1600536811030893

**Published:** 2011-08-17

**Authors:** Tao Li, Li-Rong Tang, Lin Yang, Biao Huang

**Affiliations:** a School of Life Sciences, Fujian Agriculture and Forestry University, Fuzhou, Fujian 350002, People’s Republic of China; b Material Engineering College, Fujian Agriculture and Forestry University, Fuzhou, Fujian 350002, People’s Republic of China

## Abstract

The title compound, [Zn(C_8_H_7_O_4_)_2_(H_2_O)_2_], has been synthesized by hydro­thermal methods. The Zn^II^ atom, whose symmetry element is a twofold axis, is four coordinated by two O atoms from 5-meth­oxy­salicylate anions and two aqua O atoms in a distorted tetra­hedral geometry. In the crystal, mol­ecules are linked into a layer by O—H⋯O hydrogen bonds, which stabilize the packing.

## Related literature

For coordination polymers constructed by hydrogen bonds, see: Li *et al.* (2006 ▶); Jiang *et al.* (2011 ▶). For the structure of the complex with 5-meth­oxy­salicylate ligands and its analogues, see: Púčeková-Repická *et al.* (2007 ▶); Valigura *et al.* (2006 ▶).
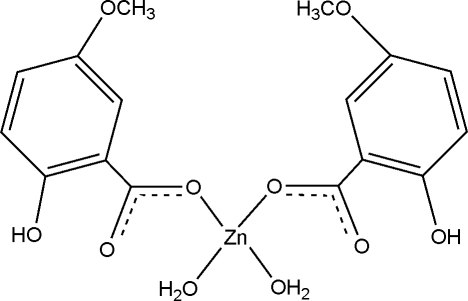


## Experimental

### 

#### Crystal data

[Zn(C_8_H_7_O_4_)_2_(H_2_O)_2_]
*M* *_r_* = 435.67
Monoclinic, 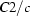

*a* = 25.113 (4) Å
*b* = 5.5065 (6) Å
*c* = 12.648 (3) Å
β = 97.845 (12)°
*V* = 1732.7 (5) Å^3^
*Z* = 4
Mo *K*α radiation
μ = 1.47 mm^−1^
*T* = 173 K
0.20 × 0.20 × 0.20 mm

#### Data collection

Rigaku Mercury CCD/AFC diffractometer
Absorption correction: multi-scan (*CrystalClear*; Rigaku, 2007 ▶) *T*_min_ = 0.745, *T*_max_ = 0.752
6403 measured reflections
1986 independent reflections
1837 reflections with *I* > 2σ(*I*)
*R*_int_ = 0.027

#### Refinement

*R*[*F* ^2^ > 2σ(*F* ^2^)] = 0.030
*wR*(*F* ^2^) = 0.082
*S* = 1.08
1986 reflections
136 parameters
H atoms treated by a mixture of independent and constrained refinement
Δρ_max_ = 0.27 e Å^−3^
Δρ_min_ = −0.69 e Å^−3^

### 

Data collection: *CrystalClear* (Rigaku, 2007 ▶); cell refinement: *CrystalClear*; data reduction: *CrystalClear*; program(s) used to solve structure: *SHELXS97* (Sheldrick, 2008 ▶); program(s) used to refine structure: *SHELXL97* (Sheldrick, 2008 ▶); molecular graphics: *SHELXTL* (Sheldrick, 2008 ▶) and *DIAMOND* (Brandenburg, 2005) ▶; software used to prepare material for publication: *SHELXTL*.

## Supplementary Material

Crystal structure: contains datablock(s) global, I. DOI: 10.1107/S1600536811030893/zk2017sup1.cif

Structure factors: contains datablock(s) I. DOI: 10.1107/S1600536811030893/zk2017Isup2.hkl

Additional supplementary materials:  crystallographic information; 3D view; checkCIF report

## Figures and Tables

**Table 1:** Hydrogen-bond geometry (Å, °)

*D*—H⋯*A*	*D*—H	H⋯*A*	*D*⋯*A*	*D*—H⋯*A*
O3—H1⋯O1	0.87 (3)	1.76 (3)	2.5771 (19)	155 (2)
O4—H4*A*⋯O2^i^	0.73 (3)	1.93 (3)	2.643 (2)	169 (3)
O4—H4*B*⋯O5^ii^	0.84 (3)	1.97 (3)	2.790 (2)	167 (3)

Symmetry codes: (i) 
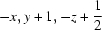
; (ii) 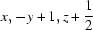.

**Fig. 1.**
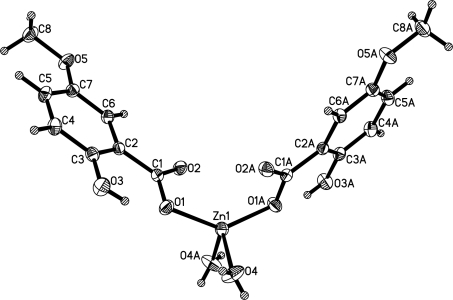
Displacement ellipsoids are drawn at the 30% probability level. (-x, y, -z + 1/2)

**Fig. 2.**
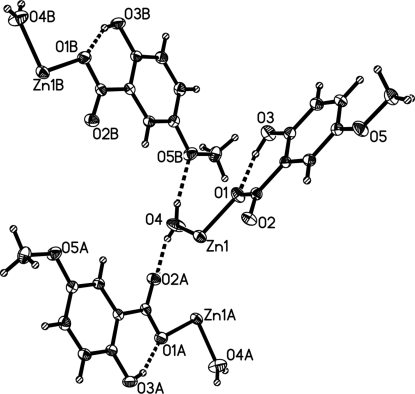
The intramolecular and intermoleclar hydrogen bonds (the dashed lines) in title compound. Symmetry codes: (A)x, -y + 2, z + 1/2; (B)x, -y + 1, z + 1/2.

**Fig. 3.**
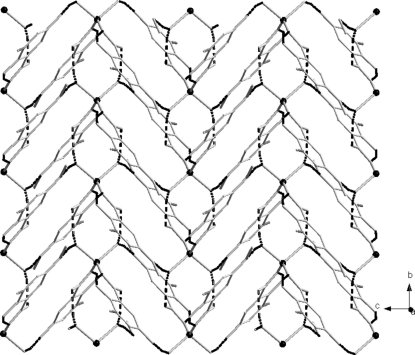
The 2-D layer of title compound by O—H—O interactions (black dash lines) viewed from a direction.

## supplementary crystallographic information

### Comment

Generally, the self-assembly of inorganic metal species and organic ligands can be achieved by the use of noncovalent contacts. Among the noncovalent contacts, hydrogen bonds have the great influence in determining the geometry of the obtained metal complexes (Li *et al.* 2006; Jiang *et al.* 2011). In the other hand, the reactions of transition metal ions with 5-MeOsal ligands and its analogues are relatively rare (Valigura *et al.*, 2006; Púčeková-Repická *et al.*,2007). In this paper we present results of hydrothermal reactions of ZnCl_2_ and 5-MeOsal ligand. Title Compound was isolated as molecular complex and the intermolecular O—H—O hydrogen bonds in (I) together with intramolecular O—H—O hydrogen bonds promote the molecules into two-dimensional structure.

A single-crystal X-ray diffraction study of (I) reveals a discrete coordination complex that crystallizes in the space group *C*2/*c*. Coordination of the Zn atoms in (I) is a slightly distorted tetrahedral geometry with each Zn atom bonding to four oxygen atoms, in which two from the monodentate 5-MeOSal ligands and two from the waters (Fig.1). Further inspection into the structure reveals that there are O—H—O intramolecular and intermolecular hydrogen bonds. (Table 1). As described in Figure 2, the intramolecular hydrogen bonds from hydroxyl hydrogen atom of the 5-MeOsal anions to the coordinated carboxylate oxygen atoms O3—H—O1 with the distance of 2.577 (1) Å, then create six-memberedrings(O_2_C_3_H), and stabilize the structure. The mean deviation from the planes C(2)—C(3)—C(4)—C(5)—C(6)-(7), and O(1)—H(13)—O(3)—C(3)—C(2)—C(1)are 0.0032 and 0.0229 Å,respectively. Furthermore the dihedral angles between planes is 0.9°, indicating a planar structure for this ligand. Furthermore the uncoordinated oxygen atoms of the carboxyl group and methoxy group of the 5-MeOsal anions(O2, O5) are hydrogen bonded with the hydrogen atoms of coordinated water with the distances of 2.643 (2) and 2.790 (2) Å, respectively. As a result every coordinated water molecule is connected by two oxygen atoms through hydrogen bonds and the isolated units are to be form a 2-D layer. A prospective view of the structure packing from *a* direction is presented in Fig. 3.

### Experimental

The title compound was hydrothermally synthesized under autogenous pressure. A mixture of ZnCl_2_ (68 mg, 0.5 mmol), 5-MeOsal (84 mg, 0.5 mmol), and H_2_O (6 ml) was sealed in a stainless reactor with Teflon liner, which was heated to 358 K for two days. After slow cooling to room temperature, prism pale yellow crystals were obtained as a major phase by filtration, which were washed with distilled water, and finally dried in air(65% yield).Anal. calc. for C_16_H_18_O_10_Zn: C, 44.11; H, 4.16; O, 36.72%; Found: C, 44.32; H, 4.15; O, 36.23%. IR (KBr pellet):3448(*w*), 3290(*w*), 2839(*w*), 2615(*w*), 1661(*s*), 1616(*s*), 1486(*s*), 1428(*s*), 1223(*s*), 1188(*s*), 1033(*m*), 824(*m*), 788(*m*), 761(*m*), 678(*m*), 555(*m*), 464(*w*).

### Refinement

All the hydrogen atoms were discernible in the difference electron density maps. However, the H atoms were situated into idealized positions and constrained by the riding model approximation: *O*—H_hydroxyl_ = 0.869, C_aryl_—H_aryl_ = 0.95, C_methyl_—H_methyl_ = 0.98 and *U*_iso_H_aryl_ = 1.2*U*_eq_(C), *U*_iso_H_methyl_ = 1.5*U*_eq_(C). The highest electron-density peak is situated 1.2 Å from Zn1 and the deepest hole 0.78 Å from Zn1.

### Crystal data

**Table:**

[Zn(C_8_H_7_O_4_)_2_(H_2_O)_2_]	*F*(000) = 896
*M**_r_* = 435.67	*D*_x_ = 1.670 Mg m^−^^3^
Monoclinic, *C*2/*c*	Mo *K*α radiation, λ = 0.71073 Å
Hall symbol: -C 2yc	Cell parameters from 2689 reflections
*a* = 25.113 (4) Å	θ = 2.1–27.5°
*b* = 5.5065 (6) Å	µ = 1.47 mm^−^^1^
*c* = 12.648 (3) Å	*T* = 173 K
β = 97.845 (12)°	Prism, pale yellow
*V* = 1732.7 (5) Å^3^	0.20 × 0.20 × 0.20 mm
*Z* = 4	

### Data collection

**Table:**

Rigaku Mercury CCD/AFC diffractometer	1986 independent reflections
Radiation source: fine-focus sealed tube	1837 reflections with *I* > 2σ(*I*)
graphite	*R*_int_ = 0.027
φ and ω scans	θ_max_ = 27.5°, θ_min_ = 3.3°
Absorption correction: multi-scan (*CrystalClear*; Rigaku, 2007)	*h* = −31→32
*T*_min_ = 0.745, *T*_max_ = 0.752	*k* = −7→7
6403 measured reflections	*l* = −16→15

### Refinement

**Table:**

Refinement on *F*^2^	Primary atom site location: structure-invariant direct methods
Least-squares matrix: full	Secondary atom site location: difference Fourier map
*R*[*F*^2^ > 2σ(*F*^2^)] = 0.030	Hydrogen site location: inferred from neighbouring sites
*wR*(*F*^2^) = 0.082	H atoms treated by a mixture of independent and constrained refinement
*S* = 1.08	*w* = 1/[σ^2^(*F*_o_^2^) + (0.0494*P*)^2^] where *P* = (*F*_o_^2^ + 2*F*_c_^2^)/3
1986 reflections	(Δ/σ)_max_ = 0.001
136 parameters	Δρ_max_ = 0.27 e Å^−^^3^
0 restraints	Δρ_min_ = −0.69 e Å^−^^3^

### Special details

**Table:**

Geometry. All e.s.d.'s (except the e.s.d. in the dihedral angle between two l.s. planes) are estimated using the full covariance matrix. The cell e.s.d.'s are taken into account individually in the estimation of e.s.d.'s in distances, angles and torsion angles; correlations between e.s.d.'s in cell parameters are only used when they are defined by crystal symmetry. An approximate (isotropic) treatment of cell e.s.d.'s is used for estimating e.s.d.'s involving l.s. planes.
Refinement. Refinement of *F*^2^ against ALL reflections. The weighted *R*-factor *wR* and goodness of fit *S* are based on *F*^2^, conventional *R*-factors *R* are based on *F*, with *F* set to zero for negative *F*^2^. The threshold expression of *F*^2^ > σ(*F*^2^) is used only for calculating *R*-factors(gt) *etc*. and is not relevant to the choice of reflections for refinement. *R*-factors based on *F*^2^ are statistically about twice as large as those based on *F*, and *R*- factors based on ALL data will be even larger.

### Fractional atomic coordinates and isotropic or equivalent isotropic displacement parameters (Å^2^)

**Table:**

	*x*	*y*	*z*	*U*_iso_*/*U*_eq_	
Zn1	0.0000	0.94430 (5)	0.2500	0.03390 (13)	
O1	0.07205 (5)	0.8141 (2)	0.23289 (10)	0.0398 (3)	
O2	0.01520 (5)	0.6023 (2)	0.12225 (11)	0.0379 (3)	
O3	0.17146 (5)	0.6892 (3)	0.27805 (10)	0.0433 (3)	
O4	0.02830 (7)	1.1674 (3)	0.36768 (14)	0.0556 (4)	
O5	0.12868 (6)	−0.0192 (3)	−0.03081 (11)	0.0432 (3)	
C1	0.06216 (6)	0.6401 (3)	0.16527 (12)	0.0294 (3)	
C2	0.10816 (6)	0.4881 (3)	0.14311 (13)	0.0269 (3)	
C3	0.15997 (7)	0.5201 (3)	0.19989 (13)	0.0301 (3)	
C4	0.20140 (7)	0.3720 (4)	0.17747 (14)	0.0358 (4)	
H4	0.2364	0.3946	0.2153	0.043*	
C5	0.19295 (7)	0.1916 (3)	0.10108 (14)	0.0355 (4)	
H5	0.2219	0.0912	0.0866	0.043*	
C6	0.09982 (6)	0.3065 (3)	0.06567 (12)	0.0297 (3)	
H6	0.0651	0.2848	0.0266	0.036*	
C7	0.14159 (7)	0.1582 (3)	0.04532 (12)	0.0307 (3)	
C8	0.17070 (8)	−0.1737 (4)	−0.05639 (16)	0.0470 (5)	
H8A	0.1989	−0.0751	−0.0818	0.070*	
H8B	0.1562	−0.2881	−0.1123	0.070*	
H8C	0.1859	−0.2639	0.0074	0.070*	
H1	0.1412 (11)	0.767 (5)	0.275 (2)	0.065 (8)*	
H4A	0.0164 (10)	1.283 (5)	0.378 (2)	0.052 (7)*	
H4B	0.0570 (12)	1.135 (6)	0.407 (2)	0.081 (9)*	

### Atomic displacement parameters (Å^2^)

**Table:**

	*U*^11^	*U*^22^	*U*^33^	*U*^12^	*U*^13^	*U*^23^
Zn1	0.02475 (17)	0.03398 (19)	0.0432 (2)	0.000	0.00526 (12)	0.000
O1	0.0334 (6)	0.0375 (7)	0.0492 (7)	−0.0006 (5)	0.0087 (5)	−0.0142 (5)
O2	0.0256 (6)	0.0363 (6)	0.0511 (7)	0.0028 (5)	0.0025 (5)	−0.0042 (5)
O3	0.0319 (7)	0.0497 (8)	0.0462 (7)	−0.0039 (6)	−0.0015 (6)	−0.0164 (6)
O4	0.0486 (9)	0.0465 (9)	0.0660 (10)	0.0189 (8)	−0.0124 (8)	−0.0210 (8)
O5	0.0393 (7)	0.0429 (7)	0.0464 (8)	0.0083 (6)	0.0020 (6)	−0.0165 (6)
C1	0.0283 (8)	0.0280 (8)	0.0330 (8)	−0.0011 (7)	0.0081 (6)	0.0022 (6)
C2	0.0244 (8)	0.0279 (7)	0.0288 (8)	−0.0005 (7)	0.0051 (6)	0.0021 (6)
C3	0.0277 (8)	0.0338 (8)	0.0287 (8)	−0.0039 (7)	0.0027 (6)	−0.0002 (6)
C4	0.0237 (8)	0.0458 (9)	0.0366 (8)	0.0012 (8)	−0.0002 (7)	0.0002 (7)
C5	0.0279 (8)	0.0418 (9)	0.0372 (8)	0.0090 (7)	0.0053 (7)	0.0016 (7)
C6	0.0251 (7)	0.0330 (8)	0.0302 (7)	−0.0006 (6)	0.0015 (6)	−0.0004 (6)
C7	0.0321 (8)	0.0309 (8)	0.0291 (7)	0.0017 (7)	0.0045 (6)	−0.0003 (6)
C8	0.0519 (12)	0.0448 (11)	0.0466 (10)	0.0112 (9)	0.0155 (9)	−0.0063 (8)

### Geometric parameters (Å, °)

**Table:**

Zn1—O4	1.9852 (15)	C2—C6	1.395 (2)
Zn1—O4^i^	1.9852 (15)	C2—C3	1.409 (2)
Zn1—O1	1.9854 (13)	C3—C4	1.382 (2)
Zn1—O1^i^	1.9854 (13)	C4—C5	1.382 (3)
O1—C1	1.286 (2)	C4—H4	0.9500
O2—C1	1.247 (2)	C5—C7	1.395 (2)
O3—C3	1.360 (2)	C5—H5	0.9500
O3—H1	0.87 (3)	C6—C7	1.381 (2)
O4—H4A	0.73 (3)	C6—H6	0.9500
O4—H4B	0.84 (3)	C8—H8A	0.9800
O5—C7	1.379 (2)	C8—H8B	0.9800
O5—C8	1.427 (2)	C8—H8C	0.9800
C1—C2	1.484 (2)		
			
O4—Zn1—O4^i^	103.54 (11)	C4—C3—C2	119.37 (16)
O4—Zn1—O1	93.83 (6)	C3—C4—C5	121.33 (15)
O4^i^—Zn1—O1	112.34 (7)	C3—C4—H4	119.3
O4—Zn1—O1^i^	112.34 (7)	C5—C4—H4	119.3
O4^i^—Zn1—O1^i^	93.83 (6)	C4—C5—C7	119.43 (16)
O1—Zn1—O1^i^	137.66 (7)	C4—C5—H5	120.3
C1—O1—Zn1	104.20 (10)	C7—C5—H5	120.3
C3—O3—H1	102.2 (17)	C7—C6—C2	120.63 (14)
Zn1—O4—H4A	125 (2)	C7—C6—H6	119.7
Zn1—O4—H4B	120 (2)	C2—C6—H6	119.7
H4A—O4—H4B	115 (3)	O5—C7—C6	115.91 (14)
C7—O5—C8	118.04 (15)	O5—C7—C5	124.02 (15)
O2—C1—O1	120.00 (15)	C6—C7—C5	120.07 (15)
O2—C1—C2	122.53 (15)	O5—C8—H8A	109.5
O1—C1—C2	117.46 (14)	O5—C8—H8B	109.5
C6—C2—C3	119.16 (15)	H8A—C8—H8B	109.5
C6—C2—C1	119.38 (14)	O5—C8—H8C	109.5
C3—C2—C1	121.45 (15)	H8A—C8—H8C	109.5
O3—C3—C4	117.55 (15)	H8B—C8—H8C	109.5
O3—C3—C2	123.07 (16)		

Symmetry codes: (i) −*x*, *y*, −*z*+1/2.

### Hydrogen-bond geometry (Å, °)

**Table:**

*D*—H···*A*	*D*—H	H···*A*	*D*···*A*	*D*—H···*A*
O3—H1···O1	0.87 (3)	1.76 (3)	2.5771 (19)	155 (2)
O4—H4A···O2^ii^	0.73 (3)	1.93 (3)	2.643 (2)	169 (3)
O4—H4B···O5^iii^	0.84 (3)	1.97 (3)	2.790 (2)	167 (3)

Symmetry codes: (ii) −*x*, *y*+1, −*z*+1/2; (iii) *x*, −*y*+1, *z*+1/2.
